# Associations between physical activity levels and chronic lung diseases in middle-aged and older adults in China: A CHARLS-based study

**DOI:** 10.1371/journal.pone.0354445

**Published:** 2026-09-08

**Authors:** Zhaoyin Su, Long Pu, Rui Huang, Yifu Zhu, Yifeng Lin, Yuli He, Shuangwei Mao, Weitao Wang, Weihan Kang, Yao Liu, Yatao Liu, Rubing Lin, Nerlich Michael

**Affiliations:** 1 The First School of Clinical Medicine, Lanzhou University, Lanzhou, China; 2 School of Basic Medical Sciences, Lanzhou University, Lanzhou, China; 3 The Second School of Clinical Medicine, Lanzhou University, Lanzhou, China; 4 School of Public Health, Lanzhou University, Lanzhou, China; 5 Department of Anesthesia, First Hospital of Lanzhou University, Lanzhou, China; 6 Department of Orthopedics, Shenzhen Children’s Hospital, Shenzhen, China; 7 Department of Trauma Surgery, University Hospital Regensburg, Regensburg, Germany; Soochow University, CHINA

## Abstract

**Background:**

Chronic lung diseases (CLDs) pose a significant public health challenge globally, particularly among aging populations. Physical activity (PA) is a modifiable lifestyle factor with potential benefits for respiratory health. This study aimed to investigate the association between PA levels and the odds of CLDs among middle-aged and older adults in China.

**Method:**

Data were derived from the 2020 China Health and Retirement Longitudinal Study (CHARLS). A total of 8,143 participants aged 45 years and older were included. Multivariable logistic regression and subgroup analyses were performed to examine the association between PA and CLDs.

**Result:**

The overall prevalence of CLDs was 18.11% (1,475/8,143). In the multivariable model, high-intensity physical activity was associated with lower odds of CLDs compared to low-intensity activity (OR=0.86, 95% CI: 0.74–0.99, P = 0.039), while moderate-intensity activity showed a trend but was not significant (OR=0.87, 95% CI: 0.74–1.02, P = 0.085). Subgroup analyses revealed a significant interaction by sex and age (P for interaction <0.05). The inverse association was significant among males (moderate: OR=0.75; high: OR=0.74) and among participants aged ≥65 years (high-intensity: OR=0.80, 95% CI: 0.68–0.94, P = 0.008), but not among females.

**Conclusion:**

This cross-sectional analysis found that high-intensity physical activity was independently associated with lower odds of CLDs in Chinese adults aged 45 and older, with the association varying by age and sex. Promoting regular physical activity, particularly in older adults and middle-aged men, may be a valuable public health strategy for respiratory health in China’s aging population. Further longitudinal studies are needed to confirm these findings.

## 1. Introduction

Chronic lung diseases (CLDs) is an umbrella term that refers to a group of long-term respiratory conditions that affect lung function and can lead to persistent breathing difficulties [1]. It encompasses chronic bronchitis, emphysema, pulmonary heart disease, asthma, and other related conditions [2,3]. In the world, over 545 million people suffer from CLDs, leading to over 4 million deaths annually, making it one of the leading causes of global mortality [4]. Chronic obstructive pulmonary disease (COPD) and asthma are two of the most common and impactful CLDs, affecting millions of people worldwide [5]. COPD is characterized by persistent respiratory symptoms and airflow limitation caused by exposure to harmful particles or gases [6], while asthma is a heterogeneous disease marked by chronic airway inflammation [7]. These conditions lead to disability-adjusted life years and reduced quality of life, as well as significant economic costs from healthcare expenses and lost productivity [5,8,9].

The pathogenesis of CLDs involves a complex interplay of genetic factors and environmental exposures, resulting in chronic inflammation, lung structural changes, and declining respiratory function [10]. This can cause symptoms like difficulty breathing, fatigue, anxiety, depression, and fear, limiting individuals’ ability to exercise and perform daily tasks, reducing their quality of life, and raising the chances of hospitalization and death [9]. The annual treatment costs for chronic lung diseases amount to billions of dollars, contributing to the overall burden of noncommunicable diseases in society [9,11,12]. which is expected to persist as a primary societal burden in the foreseeable future [13].

The global demographic shift towards an aging population further exacerbates the challenge posed by chronic diseases, including CLDs. [14]. Middle-aged and older individuals are disproportionately affected by these conditions due to a combination of factors, including cumulative lifetime exposure to risk factors such as tobacco smoke and air pollution, age-related physiological changes in lung structure and function (immunosenescence), and the increased prevalence of comorbidities that can complicate CLDs management and outcomes [15–17]. For this demographic, CLDs often lead to a significant decline in physical functioning, increased dependency, social isolation, and a substantially reduced quality of life [18]. The burden on healthcare services also intensifies with an aging population, as older adults typically require more frequent medical consultations, emergency department visits, and hospital admissions [19].

China, as the world’s second populous nation, is experiencing an unprecedented rate of population aging [20], presenting unique and formidable public health challenges. Concurrent with this demographic transition, China bears a significant burden of chronic diseases, including CLDs [21]. Numerous nationwide surveys have shown a significant number of cases of CLDs, especially in individuals over 40 years old, due to factors like high rates of smoking, prolonged exposure to indoor and outdoor air pollution from biomass fuel and industrial emissions, and occupational risks [22–25]. The prevalence of CLDs among middle-aged and elderly individuals in China is a significant concern, as highlighted by recent research using data from the China Health and Retirement Longitudinal Study [16,21]. These conditions significantly add to the total disease burden in this age bracket, affecting health outcomes and economic well-being through higher healthcare costs and decreased ability to work and carry out daily tasks.

In the quest to mitigate the burden of CLDs, identifying modifiable lifestyle factors is of paramount importance. Physical activity (PA), defined as any bodily movement produced by skeletal muscles that results in energy expenditure, stands out as a cornerstone of preventive medicine and health promotion [26]. PA encompasses a wide spectrum of activities, including exercise [27], sports [28], active mobility [29], and recreational pursuits [30]. The health benefits of regular PA are extensive and well-documented, spanning improvements in cardiovascular health, metabolic function, musculoskeletal strength, and mental well-being [31–33]. Global guidelines consistently recommend regular PA for all age groups, with specific advice tailored for older adults to maintain functional independence and prevent chronic conditions [31,32]. For instance, the World Health Organization recommends that adults should engage in at least 150–300 minutes of moderate-intensity aerobic activity per week, or 75–150 minutes of high-intensity aerobic activity per week, or a combination of both [34].

Recently, the role of PA in respiratory health, both in the prevention and management of CLDs, has garnered increasing attention [35,36]. Mechanistically, regular PA may confer benefits to the respiratory system through several pathways. These include improvements in ventilatory efficiency, strengthening of respiratory muscles, modulation of inflammatory responses, enhancement of antioxidant defenses, and favorable effects on airway remodeling [10,37–39]. Epidemiological studies have suggested an inverse association between PA levels and the risk of developing CLDs [40]. For individuals already living with CLDs, PA is a critical component of comprehensive management [41]. Pulmonary rehabilitation programs, which have structured exercise training as a core element, have demonstrated significant improvements in exercise capacity, symptoms, health-related quality of life, and reductions in hospital admissions for patients with COPD [41–43]. Similarly, PA interventions have shown promise in improving asthma control and lung function in asthmatic patients [44]. A recent study in China highlighted that a theory-based behavior change intervention could promote PA and improve outcomes such as dyspnea, exercise capacity, and health-related quality of life in patients with COPD [45].

In this context, examining the associations between physical activity levels and the prevalence of CLDs in middle-aged and older adults becomes crucial, particularly in China, where aging populations face increasing respiratory health challenges. Recent data indicate that a considerable proportion of Chinese middle-aged and older adults do not meet recommended PA guidelines [21]. PA levels in this demographic are influenced by a complex interplay of factors, including socioeconomic status, urban versus rural residence, employment status [46,47]. Retirement, a significant life transition for older adults, can also impact PA behaviors, with some studies suggesting a potential decline in overall activity if occupational PA is not replaced by leisure-time PA [46]. While the general association between PA and reduced risk of chronic diseases, including some CLDs, is recognized [21]. There is a need for more specific evidence regarding the dose-response relationship between different levels of PA (e.g., low, moderate, high) and the odds of various CLDs, particularly within the unique demographic and socio-cultural context of China’s rapidly aging population. Large-scale, nationally representative studies are essential to provide robust evidence that can inform targeted public health interventions and clinical recommendations. Cross-sectional studies, while unable to establish causality, play a vital role in identifying associations, generating hypotheses for future longitudinal research, and providing a snapshot of the current public health landscape. The China Health and Retirement Longitudinal Study (CHARLS) offers a valuable platform for such investigations, providing comprehensive data on health status, lifestyle factors including PA, and sociodemographic characteristics of a nationally representative sample of middle-aged and older Chinese adults [16,21,46,47]. Previous research using CHARLS has already shed light on the relationship between PA and overall chronic disease prevalence [21], but a focused analysis on specific CLDs and different PA intensity levels is warranted.

Therefore, this study aims to investigate the cross-sectional association between different levels of physical activity and the odds of CLDs among middle-aged and older adults in China, utilizing data from the CHARLS. By examining this relationship in a large, nationally representative sample, this study seeks to provide valuable insights that can contribute to the development of evidence-based strategies for the prevention and management of CLDs in China’s aging population. The findings may help to refine public health messaging regarding PA, guide the design of culturally appropriate PA promotion programs, and inform clinical practice in advising older adults on lifestyle modifications to maintain respiratory health. Understanding these associations is particularly critical given the dual pressures of a rapidly aging demographic and the substantial burden of CLDs in China, highlighting an urgent need for effective preventive measures.

## 2. Materials and Methods

### 2.1. Data source and participants

**Fig 1:**
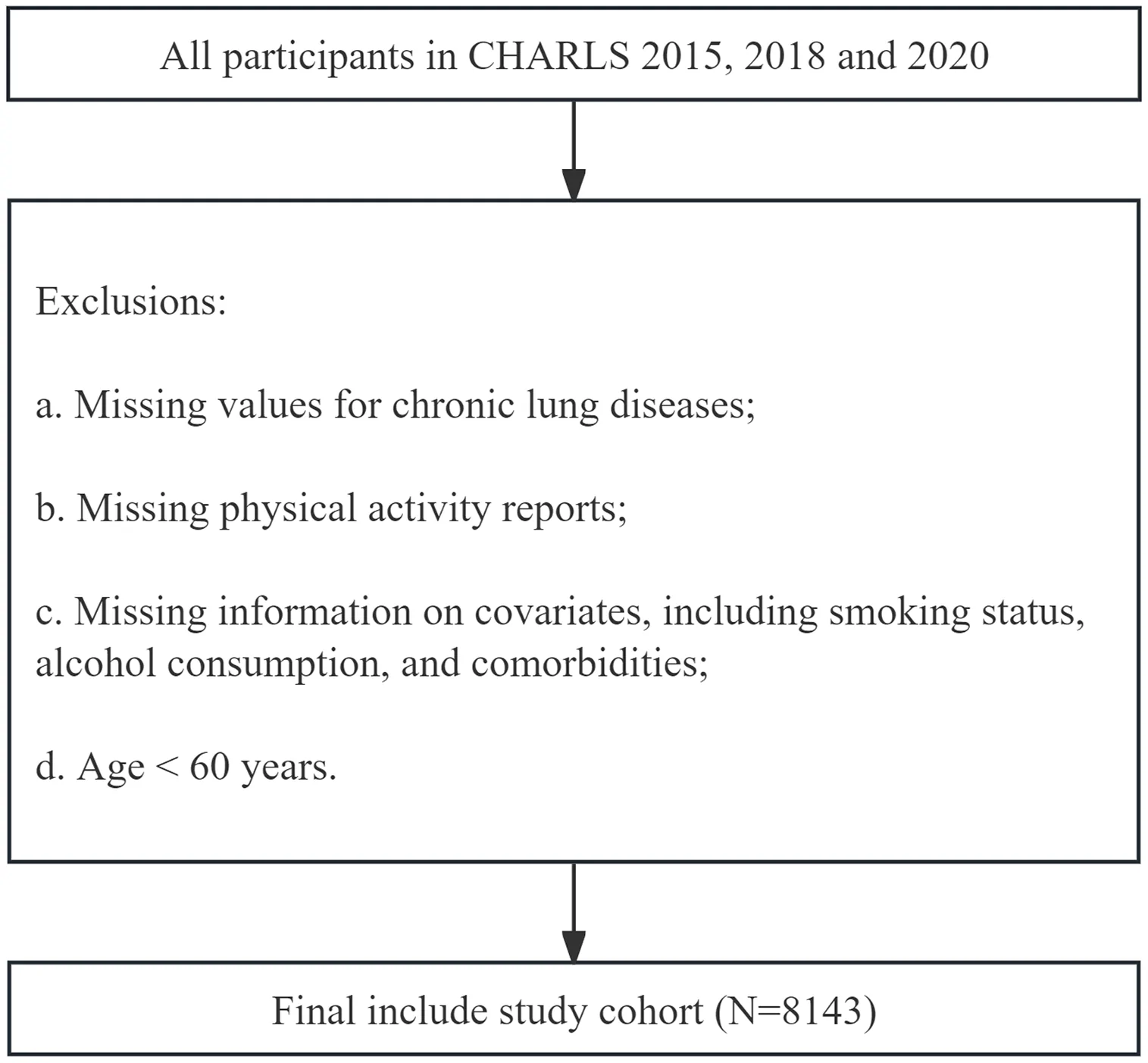
Flowchart of Study Participant Selection.

We used data from the China Health and Retirement Longitudinal Study (CHARLS) [48], which is publicly available at http://charls.pku.edu.cn. This study was approved by the Biomedical Ethics Committee of Peking University. The data were of high quality and had a large sample size, making them suitable for the analyses in this paper. We initially used data from the 2020 wave of CHARLS to identify eligible participants and assess missing information on exposure, outcome, and covariates. For participants with missing values in the 2020 wave, we supplemented these items using corresponding data from the 2018 and 2015 waves, when available. After exclusions, 8143 participants were included in the final analytic sample (Fig 1). Our research was conducted in accordance with the principles of the Helsinki Declaration. The original CHARLS study was approved by the Institutional Review Board of Peking University (IRB0000105211,015), and all participants provided informed consent before taking part in the study.

### 2.2. Ethics statement

The CHARLS study is approved by the Biomedical Ethics Review Committee of Peking University (IRB00001052–11015). As part of the field survey, every respondent who agreed to participate was asked to sign two informed consent forms. They were clearly informed about the study’s purpose, procedures, risks, and their rights, ensuring voluntariness. One copy of the form was kept by the respondent, while the other was submitted to the CHARLS office, where it was scanned and stored in PDF format for safekeeping. The CHARLS data are freely available on the Peking University Open Research Data Platform (https://charls.charlsdata.com).

### 2.3. Measures

#### 2.3.1. Assessment of physical activity level.

The daily duration of participants’ physical activity was categorized into five tiers based on questionnaire responses (0 min, 10–29 min, 30–119 min, 120–239 min, > 240 min), with median values used for calculations [49]. Physical activity was classified as follows: ① High-intensity physical activity such as mountain climbing, running, and farming; ② Moderate-intensity physical activity including brisk walking and Tai Chi; ③ Low-intensity physical activity like leisurely strolling [50]. Weekly physical activity duration was computed as the number of days of physical activity multiplied by the duration of each type of physical activity per day. Physical activity volume was quantified using metabolic equivalent (MET) [51], with MET values assigned based on the International Physical Activity Questionnaire (IPAQ) criteria: 3.3 for low-intensity, 4.0 for moderate-intensity, and 8.0 for high-intensity physical activity [52,53].

The formula for calculating the Total physical activity is as follows:

Total physical activity = (duration of High-intensity physical activity per day × number of days per week × 8.0 metabolic equivalent) + (duration of Moderate-intensity physical activity per day × number of days per week × 4.0 metabolic equivalent) + (duration of Low-intensity physical activity per day × number of days per week × 3.3 metabolic equivalent) [54,55].

Based on this score, weekly physical activity levels were classified as low-intensity (<600 METs/week), moderate-intensity (600–3000 METs/week), and high-intensity (>3000 METs/week) [56].

#### 2.3.2. Assessment of chronic lung disease.

In determining whether a participant had CLDs, both the participant and an informant familiar with the participant’s health were asked. If the participant could self-report, they provided the information directly. If the participant was unable to do so due to health conditions or other reasons, a close family member, typically the one most familiar with the participant’s health, provided the necessary information. Specifically, members of the CHARLS survey team inquired during their visits whether respondents had ever experienced or developed CLDs. If a respondent answered affirmatively and provided relevant diagnostic evidence, it was classified as the occurrence of CLDs.

#### 2.3.3. Covariates.

There are many factors affecting the prevalence of CLDs [1]. Based on previous literature and the availability of data, the following covariates were selected for adjustment in the analysis. Sociodemographic covariates included age (<65 years, ≥ 65 years), sex (male, female), education level (primary school and below, middle school, high school and above), residential area (urban, peri-urban, rural), and marital status (married, separated, divorced, widowed, single). Health-related behavior covariates included smoking status (yes, never), drinking frequency (monthly more than once, monthly less than once, never), and body mass index (BMI) categorized as underweight, normal, overweight, and obese. Comorbidities included self-reported physician-diagnosed hypertension (no, yes), hyperlipidemia (no, yes), and diabetes (no, yes). Physical activity level (low-intensity, moderate-intensity, vigorous-intensity physical activity) was treated as the main exposure rather than a covariate.

### 2.4. Statistical analysis

Data management and cleaning were performed using Stata 16.0 (StataCorp LLC, College Station, TX, USA), and statistical analyses were conducted using SPSS 27.0 (IBM Corp., Armonk, NY, USA). Categorical variables were presented as n (%). Group comparisons were performed using the chi-square test.

Variables with a p-value < 0.05 in univariable analysis were entered into multivariable logistic regression models. Two a priori nested multivariable logistic regression models were specified: Model 1 was adjusted for demographic factors (age, sex, education level, residential area, and marital status); Model 2 was additionally adjusted for lifestyle factors (smoking status, drinking frequency) and comorbidities (BMI, hypertension, hyperlipidemia, and diabetes). The Akaike information criterion (AIC) and Hosmer–Lemeshow goodness-of-fit test were used to compare model fit: AIC was 7636.1 for Model 1 and 7528.6 for Model 2, indicating better fit for Model 2; both models yielded non-significant Hosmer–Lemeshow p-values (p > 0.05), suggesting adequate model calibration. Multicollinearity among covariates was assessed using the variance inflation factor (VIF). A VIF threshold of < 5 was used to define the absence of severe multicollinearity.

Interaction terms between physical activity and age/sex were tested in the multivariable models to assess potential effect modification. Exploratory subgroup analyses stratified by age (<65 vs ≥ 65 years) and sex were also performed. A two-tailed p-value < 0.05 was considered statistically significant.

## 3. Results

### 3.1. Participant characteristics

As presented in Table 1, a total of 8,143 participants were included, comprising 3,887 males and 4,256 females (male-to-female ratio 0.91:1). The overall prevalence of CLDs was 18.11% (1,475 cases). The prevalence was significantly higher among participants aged ≥65 years and among males (both P < 0.001). Significant differences between the CLD and non-CLD groups were observed in smoking status, alcohol consumption, BMI, hypertension, hyperlipidemia, diabetes, and physical activity levels (all P < 0.001). No significant differences were found for marital status, residential area, or education level (P ≥ 0.05).

**Table 1:** Demographic characteristics of the subjects.

Variable	Total N = 8143	Non-CLDs N = 6668	CLDs N = 1475	*P*
**Age, n(%)**				<0.001
<65	2482 (30.5%)	2130 (31.9%)	352 (23.9%)	
≥65	5661 (69.5%)	4538 (68.1%)	1123 (76.1%)	
**Gender, n(%)**				<0.001
Male	3887 (47.7%)	3091 (46.4%)	796 (54.0%)	
Female	4256 (52.3%)	3577 (53.6%)	679 (46.0%)	
**Marriage, n(%)**				0.122
Married	5907 (72.5%)	4874 (73.1%)	1033 (70.0%)	
Separated	300 (3.7%)	248 (3.7%)	52 (3.5%)	
Divorced	101 (1.2%)	79 (1.2%)	22 (1.5%)	
Widowed	1789 (22.0%)	1430 (21.4%)	359 (24.3%)	
Single	46 (0.6%)	37 (0.6%)	9 (0.6%)	
**Residential area, n(%)**				0.545
Urban area	1515 (18.6%)	1228 (18.4%)	287 (19.5%)	
Peri-urban area	815 (10.0%)	675 (10.1%)	140 (9.5%)	
Rural area	5813 (71.4%)	4765 (71.5%)	1048 (71.1%)	
**Educational level, n(%)**				0.09
Primary school and below	6282 (77.1%)	5114 (76.7%)	1168 (79.2%)	
Middle school	1188 (14.6%)	986 (14.8%)	202 (13.7%)	
High school and above	673 (8.3%)	568 (8.5%)	105 (7.1%)	
**Smoking status, n(%)**				<0.001
Yes	3337 (41.0%)	2596 (38.9%)	741 (50.2%)	
Never	4806 (59.0%)	4072 (61.1%)	734 (49.8%)	
**Drinking frequency, n(%)**				0.043
Monthly more than once	1938 (23.8%)	1624 (24.4%)	314 (21.3%)	
Monthly less than once	663 (8.1%)	540 (8.1%)	123 (8.3%)	
Never	5542 (68.1%)	4504 (67.5%)	1038 (70.4%)	
**BMI, n(%)**				<0.001
Underweight	557 (6.8%)	402 (6.0%)	155 (10.5%)	
Normal	4125 (50.7%)	3377 (50.6%)	748 (50.7%)	
Overweight	2515 (30.9%)	2118 (31.8%)	397 (26.9%)	
Obese	946 (11.6%)	771 (11.6%)	175 (11.9%)	
**Hypertension, n(%)**				0.008
No	4153 (51.0%)	3447 (51.7%)	706 (47.9%)	
Yes	3990 (49.0%)	3221 (48.3%)	769 (52.1%)	
**Hyperlipidemia, n(%)**				<0.001
No	5788 (71.1%)	4813 (72.2%)	975 (66.1%)	
Yes	2355 (28.9%)	1855 (27.8%)	500 (33.9%)	
**Diabetes, n(%)**				0.002
No	6737 (82.7%)	5557 (83.3%)	1180 (80.0%)	
Yes	1406 (17.3%)	1111 (16.7%)	295 (20.0%)	
**Physical activity level, n(%)**				<0.001
Low-intensity physical activity	1719 (21.1%)	1357 (20.4%)	362 (24.5%)	
Moderate-intensity physical activity	2225 (27.3%)	1818 (27.3%)	407 (27.6%)	
High-intensity physical activity	4199 (51.6%)	3493 (52.4%)	706 (47.9%)	

CLDs, Chronic lung diseases; BMI, body mass index.

### 3.2. Univariable analysis of factors related to CLDs

**Fig 2:**
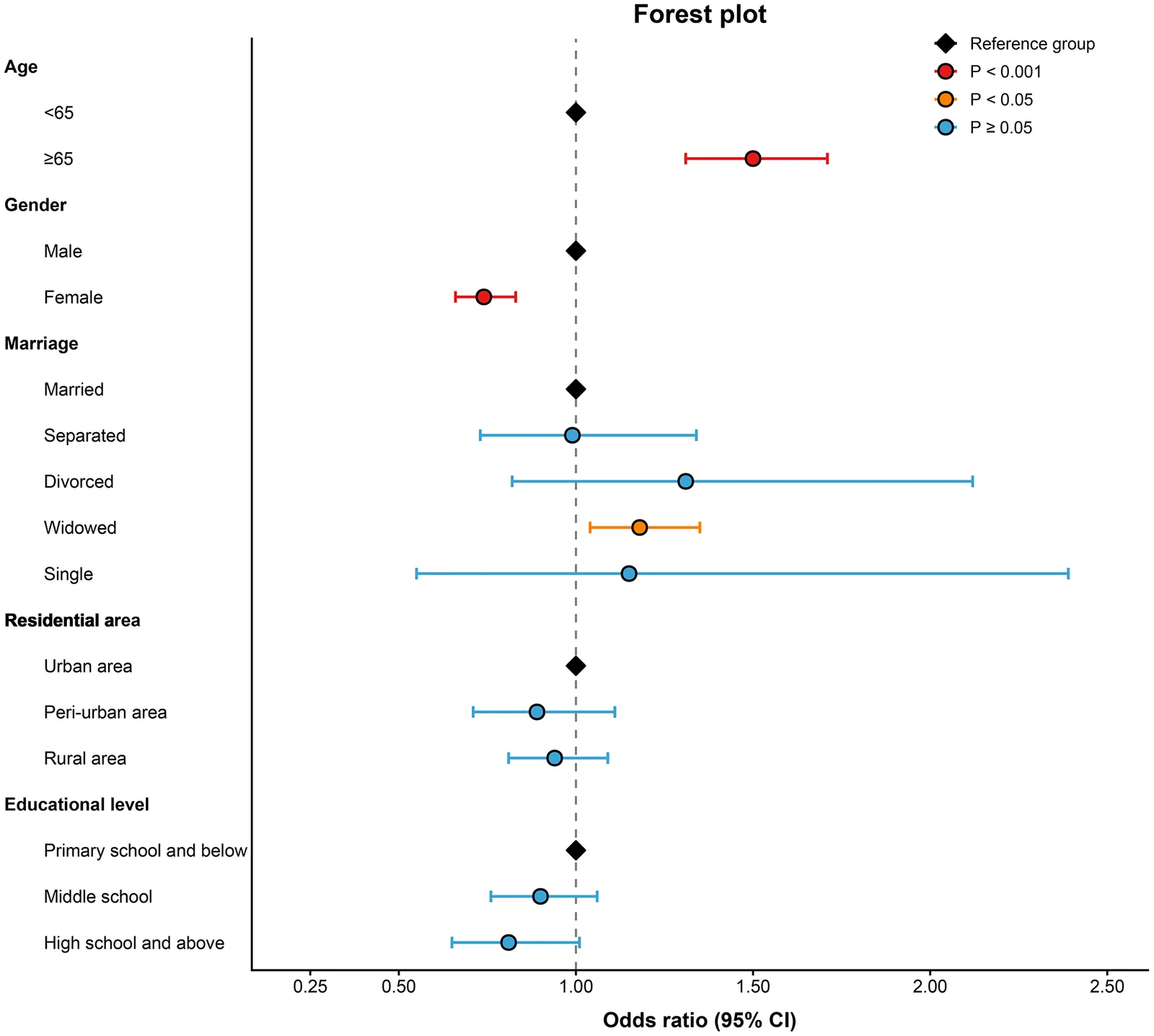
Forest plot of Univariable analysis (part 1).

**Fig 3:**
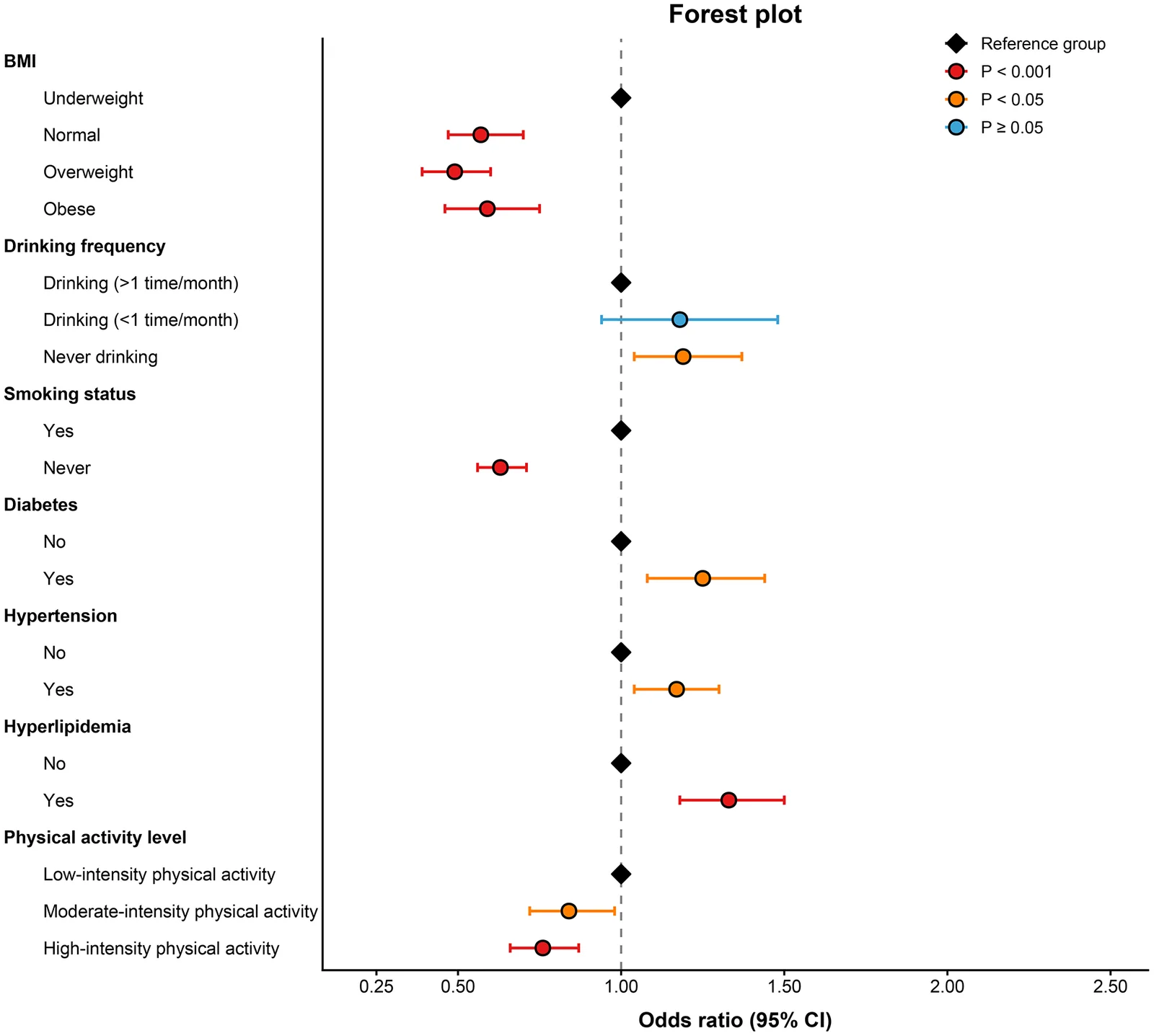
Forest plot of Univariable analysis (part 2).

As shown in Table 2 and Fig 2 and Fig 3, significant differences were observed between participants with and without CLDs in age, sex, smoking status, alcohol consumption frequency, BMI, hypertension, hyperlipidemia, diabetes, and physical activity levels (all P < 0.05), whereas no significant differences were found for marital status (except for widowed), residential area, or education level (P ≥ 0.05).

**Table 2:** Univariable analysis of the prevalence of CLDs in middle-aged and older Chinese adults.

Variable	OR	95%CI	*P*
**Age**			
<65	1.00		
≥65	1.50	1.31 ~ 1.71	**<**0.001
**Gender**			
Male	1.00		
Female	0.74	0.66 ~ 0.83	**<**0.001
**Marriage**			
Married	1.00		
Separated	0.99	0.73 ~ 1.34	0.945
Divorced	1.31	0.82 ~ 2.12	0.262
Widowed	1.18	1.04 ~ 1.35	0.013
Single	1.15	0.55 ~ 2.39	0.712
**Residential area**			
Urban area	1.00		
Peri-urban area	0.89	0.71 ~ 1.11	0.294
Rural area	0.94	0.81 ~ 1.09	0.411
**Educational level**			
Primary school and below	1.00		
Middle school	0.9	0.76 ~ 1.06	0.194
High school and above	0.81	0.65 ~ 1.01	0.057
**BMI**			
Underweight	1.00		
Normal	0.57	0.47 ~ 0.70	<0.001
Overweight	0.49	0.39 ~ 0.60	<0.001
Obese	0.59	0.46 ~ 0.75	<0.001
**Drinking frequency**			
Drinking (>1 time/month)	1.00		
Drinking (<1 time/month)	1.18	0.94 ~ 1.48	0.163
Never drinking	1.19	1.04 ~ 1.37	0.013
**Smoking status**			
Yes	1.00		
Never	0.63	0.56 ~ 0.71	<0.001
**Diabetes**			
No	1.00		
Yes	1.25	1.08 ~ 1.44	0.002
**Hypertension**			
No	1.00		
Yes	1.17	1.04 ~ 1.30	0.008
**Hyperlipidemia**			
No	1.00		
Yes	1.33	1.18 ~ 1.50	<0.001
**Physical activity level**			
Low-intensity physical activity	1.00		
Moderate-intensity physical activity	0.84	0.72 ~ 0.98	0.03
High-intensity physical activity	0.76	0.66 ~ 0.87	<0.001

CLDs, chronic lung diseases; BMI, body mass index; OR, odds ratio; CI, confidence interval

Specifically, a higher prevalence of CLDs was observed among participants aged ≥65 years and among males (both P < 0.001). Regarding lifestyle and anthropometrics, individuals who never consumed alcohol showed higher CLDs prevalence than those who drank more than once a month, whereas non-smokers and those with normal weight, overweight, or obesity showed lower CLDs prevalence compared with underweight participants. Comorbidities including diabetes, hypertension, and hyperlipidemia were more prevalent in the CLDs group. Finally, participants engaging in moderate- or high-intensity physical activity had lower likelihood of CLDs compared with those reporting low-intensity activity (P < 0.05).

### 3.3. Multivariable logistic regression analysis of factors associated with CLDs

**Fig 4:**
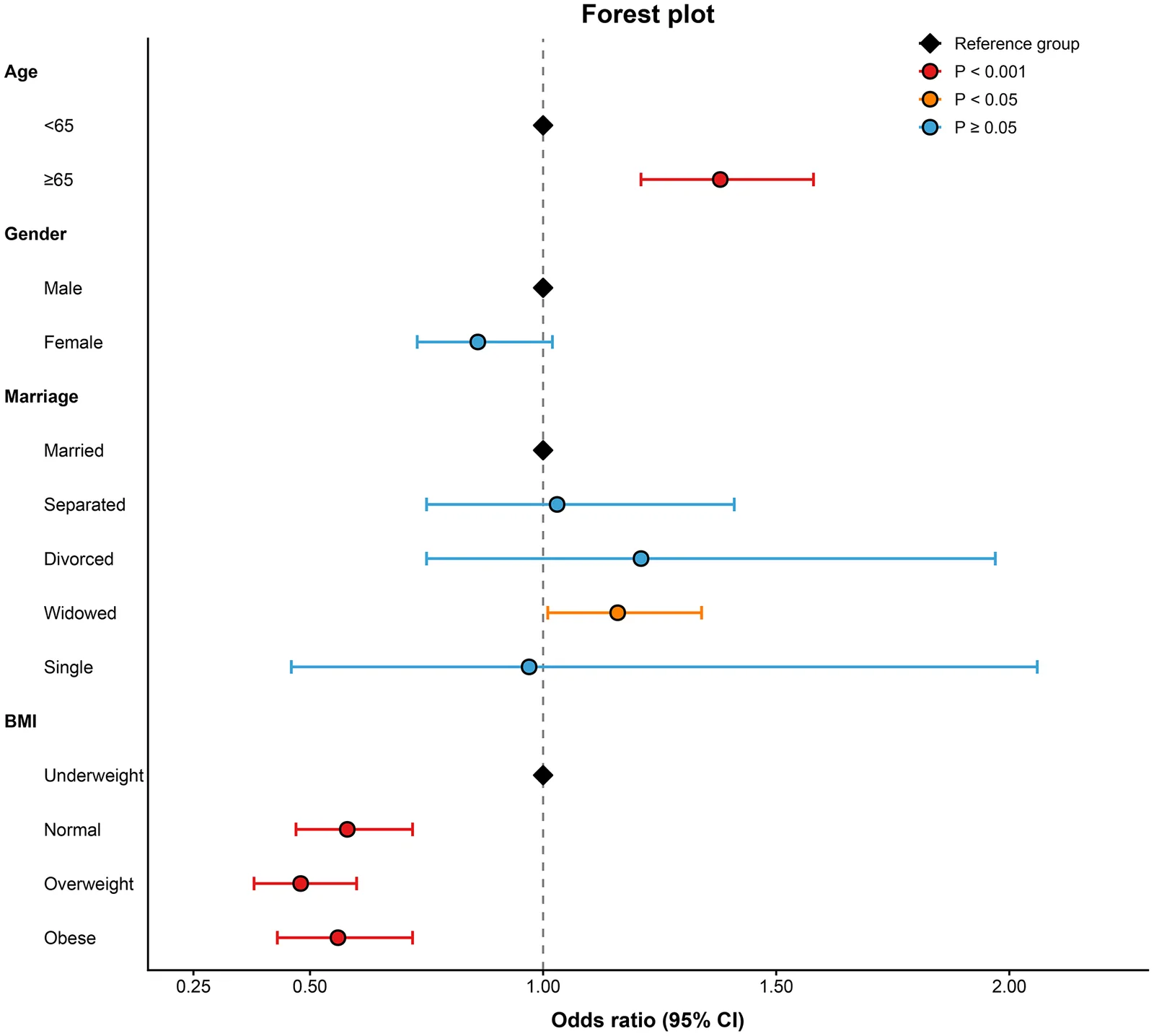
Forest plot of Multivariable analysis (part 1).

**Fig 5:**
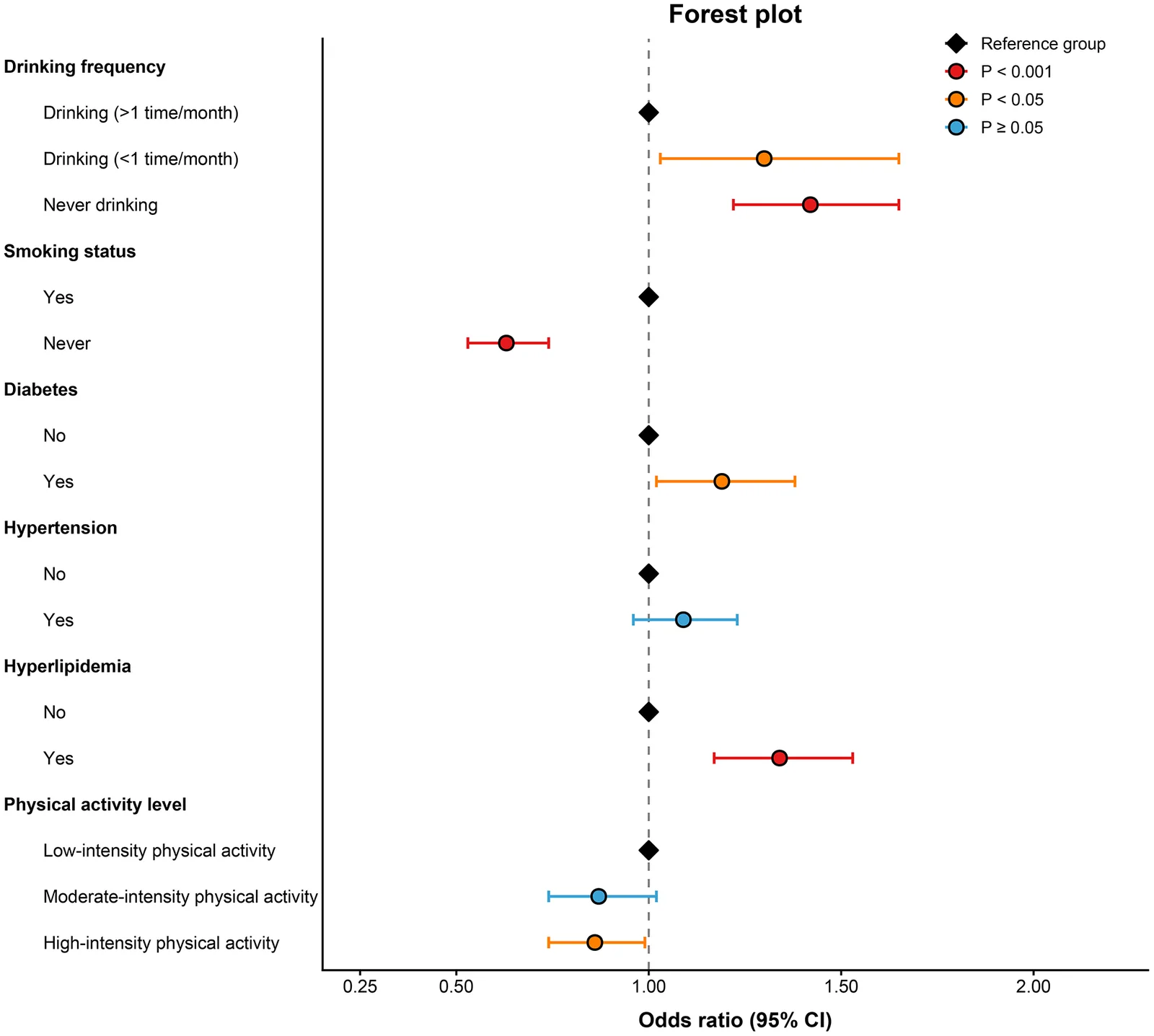
Forest plot of Multivariable analysis (part 2).

As shown in Table 3 and Fig 4 and Fig 5, the multivariable logistic regression analysis confirmed several factors independently associated with CLDs. Multicollinearity was assessed using the generalized variance inflation factor (GVIF); all GVIF^(1/(2·Df)) values were < 2.0, indicating no severe multicollinearity (S1 Appendix). Participants aged ≥65 years had significantly higher odds of CLDs than those aged <65 years (OR=1.38, 95% CI: 1.21–1.58, P < 0.001). Compared with participants who consumed alcohol more than once per month, those who never consumed alcohol showed greater odds of CLDs (OR=1.42, 95% CI: 1.22–1.65, P < 0.001).

**Table 3:** Risk factors for CLDs in middle-aged and older Chinese adults after backward regression.

Variable	OR	95%CI	*P*
**Age**			
<65	1.00		
≥65	1.38	1.21 ~ 1.58	<0.001
**Gender**			
Male	1.00		
Female	0.86	0.73 ~ 1.02	0.083
**Marriage**			
Married	1.00		
Separated	1.03	0.75 ~ 1.41	0.845
Divorced	1.21	0.75 ~ 1.97	0.437
Widowed	1.16	1.01 ~ 1.34	0.04
Single	0.97	0.46 ~ 2.06	0.943
**BMI**			
Underweight	1.00		
Normal	0.58	0.47 ~ 0.72	<0.001
Overweight	0.48	0.38 ~ 0.60	<0.001
Obese	0.56	0.43 ~ 0.72	<0.001
**Drinking frequency**			
Drinking (>1 time/month)	1.00		
Drinking (<1 time/month)	1.30	1.03 ~ 1.65	0.028
Never drinking	1.42	1.22 ~ 1.65	<0.001
**Smoking status**			
Yes	1.00		
Never	0.63	0.53 ~ 0.74	<0.001
**Diabetes**			
No	1.00		
Yes	1.19	1.02 ~ 1.38	0.031
**Hypertension**			
No	1		
Yes	1.09	0.96 ~ 1.23	0.184
**Hyperlipidemia**			
No	1.00		
Yes	1.34	1.17 ~ 1.53	<0.001
**Physical activity level**			
Low-intensity physical activity	1.00		
Moderate-intensity physical activity	0.87	0.74 ~ 1.02	0.085
High-intensity physical activity	0.86	0.74 ~ 0.99	0.039

CLDs, chronic lung diseases; BMI, body mass index; OR, odds ratio; CI, confidence interval.

Regarding BMI, participants classified as normal weight (OR=0.58, 95% CI: 0.47–0.72, P < 0.001), overweight (OR=0.48, 95% CI: 0.38–0.60, P < 0.001), or obese (OR=0.56, 95% CI: 0.43–0.72, P < 0.001) had lower odds of CLDs than underweight participants. Non-smokers also had lower odds than smokers (OR=0.63, 95% CI: 0.53–0.74, P = 0.031). In contrast, diabetes (OR=1.19, 95% CI: 1.02–1.38, P < 0.001) and hyperlipidemia (OR=1.34, 95% CI: 1.17–1.53, P < 0.001) were associated with increased odds of CLDs.

With respect to physical activity, high-intensity physical activity was associated with lower odds of CLDs compared with low-intensity activity (OR=0.86, 95% CI: 0.74–0.99, P = 0.039), whereas the association for moderate-intensity activity was not statistically significant after multivariable adjustment (OR=0.87, 95% CI: 0.74–1.02, P = 0.085). Similarly, hypertension was significantly associated with CLDs in univariable analysis but did not retain significance in the multivariable model (OR=1.09, 95% CI: 0.96–1.23, P = 0.184).

### 3.4. Sensitivity analyses and subgroup effects

**Fig 6:**
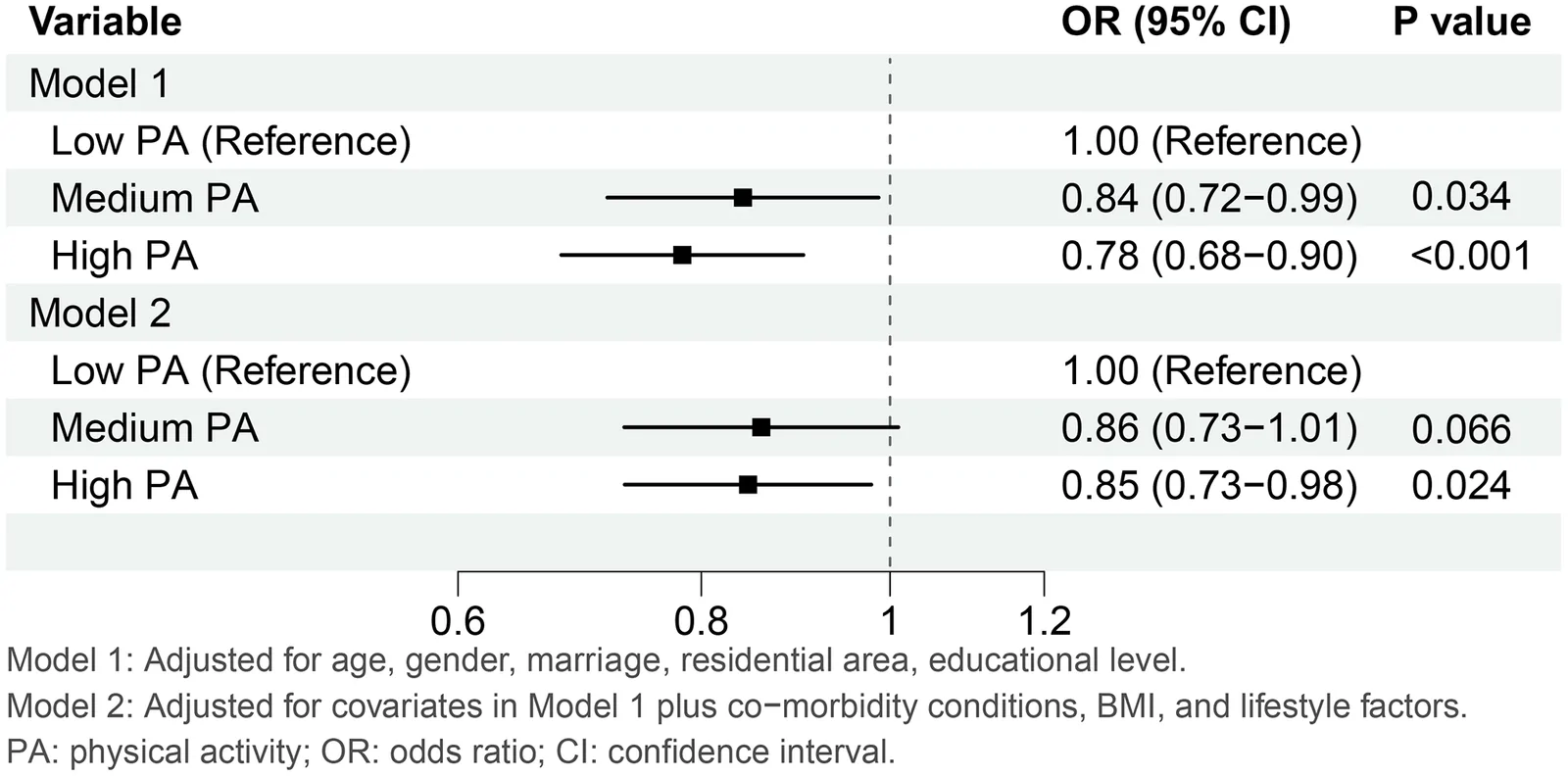
Forest plot of Sensitive analysis.

As presented in Fig 6, sensitivity analyses using nested models showed consistent results. In Model 1 (adjusted for demographics), both moderate (OR=0.84, 95% CI: 0.72–0.99) and high-intensity activity (OR=0.78, 95% CI: 0.68–0.90) were significantly associated with lower CLD odds. In Model 2 (further adjusted for BMI, comorbidities, and lifestyle), the association for moderate-intensity activity was attenuated (OR=0.86, 95% CI: 0.73–1.01, P = 0.066), whereas high-intensity activity remained significant (OR=0.85, 95% CI: 0.73–0.98, P = 0.024).

**Fig 7:**
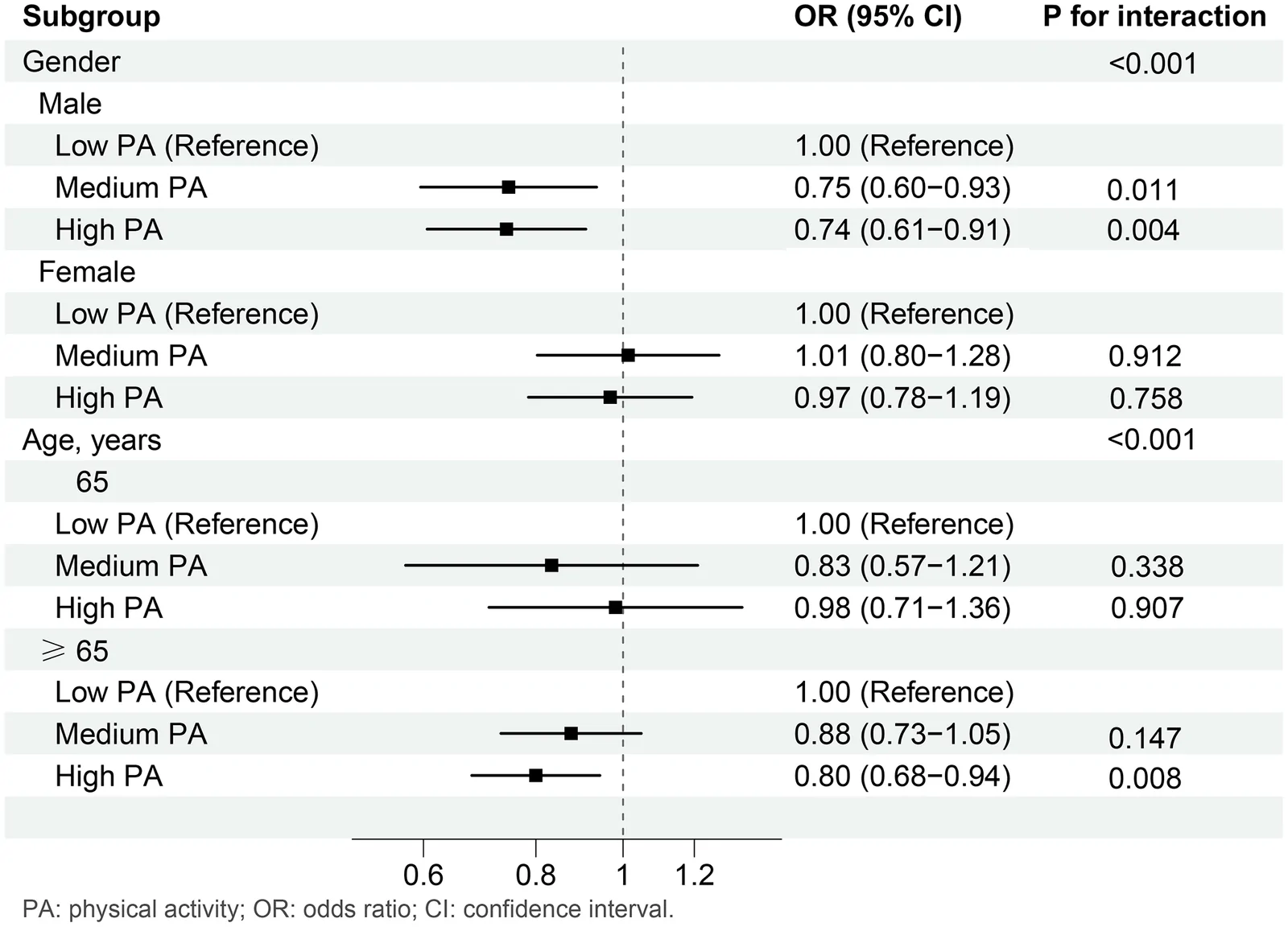
Forest plot of Subgroup analysis.

Subgroup analyses (Fig 7) revealed a significant effect modification by sex and age (P for interaction <0.05). The inverse association between physical activity and CLDs was primarily driven by the male subgroup, where both moderate (OR=0.75, 95% CI: 0.60–0.93, P = 0.011) and high-intensity activity (OR=0.74, 95% CI: 0.61–0.91, P = 0.004) showed protective effects. No significant associations were observed in females. Age-stratified analysis indicated that the protective effect of high-intensity physical activity was significant among participants aged ≥65 years (OR=0.80, 95% CI: 0.68–0.94, P = 0.008).

## 4. Discussion

Drawing on nationally representative data from the CHARLS cohort, this study identifies significant effect modification by age and gender in the association between physical activity and CLDs prevalence (P for interaction < 0.05). While high-intensity activity (≥3000 METs/week) was associated with a lower risk of CLDs in the overall population, stratified analyses revealed a more nuanced pattern: the protective effect of high-intensity exercise was primarily confined to adults aged 65 and older and male participants. Notably, among males, not only high-intensity but also moderate-intensity activity(≥600 METs/week) showed significant associations with reduced CLDs prevalence, whereas these benefits were not observed in females or younger cohorts. These findings suggest that high-intensity exercise may serve as a targeted intervention for older adults, while moderate-to-high intensity activity appears to be specifically beneficial for males. Consequently, exercise recommendations for CLDs prevention should be tailored to these specific demographic groups rather than applied as a uniform strategy across the entire middle-aged and older adult population. This supports existing literature emphasizing the health benefits of an active lifestyle [34]. and is particularly pertinent given China’s rapidly aging population and the high burden of chronic diseases [57]. In this context, promoting tailored physical activity becomes crucial not only for preventing CLDs but also for improving public health.

### 4.1. Interpretation of findings and comparison with previous research

Our study not only corroborates the established protective role of physical activity (PA) against chronic diseases but also provides novel insights into the heterogeneity of this association within the Chinese middle-aged and older adult population. Consistent with previous longitudinal evidence linking regular PA to reduced risks of hypertension, cardiovascular events, and diabetes [21,58], our findings confirm that high-intensity activity is a significant protective factor against CLDs. However, extending beyond general chronic disease literature, our results reveal a critical nuance: the beneficial effect of high-intensity activity is significantly modified by age and gender.

Specifically, while previous cohort studies, such as those conducted in Copenhagen, demonstrated that PA mitigates COPD progression and smoking-related lung function decline [59], our cross-sectional analysis suggests that this protective association is not uniform across all demographics. Unlike studies that may report a generalized benefit, our data indicate that high-intensity activity predominantly benefits adults aged 65 and older. Furthermore, our findings highlight a distinct gender disparity; notably, moderate-intensity activity was also significantly associated with lower CLDs prevalence among male participants, a specificity not observed in the female subgroup.

In the context of the CHARLS dataset, these results address a significant knowledge gap. Although prior analyses of CHARLS have established links between PA and improved cardiovascular health [60], mental health [61], and cognitive function [56], this study is among the first to specifically delineate the age and gender interactions in the context of CLDs. Given that a substantial proportion of Chinese adults fail to meet PA guidelines [21], these findings underscore the urgent need for targeted interventions. Rather than a “one-size-fits-all” approach, public health strategies should consider tailored exercise recommendations, particularly promoting moderate-to-high intensity activities for older adults and middle-aged men to effectively combat the growing burden of chronic respiratory diseases [57,62].

### 4.2. Potential biological mechanisms

Several plausible biological mechanisms may explain the inverse association between physical activity and CLDs, particularly among older adults and males.

First, regular physical activity exerts potent systemic anti-inflammatory effects [63]. Chronic low-grade inflammation is a hallmark of CLDs pathogenesis [64,65]; exercise can modulate inflammatory pathways by reducing circulating levels of pro-inflammatory cytokines (e.g., IL-6, TNF-α) and upregulating anti-inflammatory mediators [66–69]. This “anti-inflammatory environment” may mitigate the progression of airway remodeling and lung function decline [66,67].

Second, physical activity counteracts age-related declines in musculoskeletal health. Sarcopenia (age-related muscle loss) and weakened respiratory muscles contribute to dyspnea and reduced ventilatory efficiency in older adults [70,71]. Exercise enhances the strength and endurance of the diaphragm and intercostal muscles, thereby improving gas exchange and reducing the work of breathing—benefits that are particularly vital for the aging respiratory system [72,73].

Third, exercise bolsters immune surveillance and metabolic health. Physical activity improves immune cell trafficking and reduces the frequency of respiratory infections, which are common triggers for CLDs exacerbations [67,69,74,75]. Furthermore, physical activity is crucial for weight management [76]. By reducing visceral adiposity, exercise decreases the mechanical load on the diaphragm and lowers the systemic inflammatory burden originating from adipose tissue, thereby improving overall respiratory mechanics [77–79]. These physiological adaptations may explain why tailored exercise interventions are especially effective in specific demographic subgroups, such as the elderly and males, who often present with distinct metabolic and muscular profiles.

### 4.3. Strengths and limitations

The primary strength of this study is the use of a large, nationally representative sample of middle-aged and older Chinese adults from CHARLS, which enhances the generalizability of our findings to this rapidly growing population. The CHARLS dataset also provides comprehensive information, allowing us to adjust for a wide array of potential confounders, including sociodemographic characteristics, lifestyle factors (e.g., smoking and alcohol use), and comorbidities [48]. In addition, physical activity was assessed using a questionnaire based on the internationally validated IPAQ framework, which facilitates comparison with other studies [80].

However, several limitations must be acknowledged. The most significant is the cross-sectional design, which precludes any inference of causality. It is impossible to determine whether low physical activity is a risk factor for CLDs or if the presence of CLD and its associated symptoms (e.g., dyspnea, fatigue) leads to a reduction in physical activity—a classic case of reverse causality. Indeed, studies have clearly shown that patients with established COPD are markedly less active than their healthy peers [81].

Second, our study relied on self-reported data for both physical activity and CLDs diagnosis. Self-reported physical activity is prone to recall and social desirability biases and tends to overestimate actual activity levels when compared to objective measures like accelerometers [82]. While the CLDs variable was based on self-reported physician diagnoses, which is more reliable than self-diagnosis, it is still subject to misclassification and may not be as accurate as data from medical records or clinical examinations.

Third, the CLDs category in CHARLS is broad, encompassing conditions such as asthma, chronic bronchitis, and emphysema. The etiology and pathophysiology of these diseases differ, and their individual associations with physical activity may vary. This suggesting that future research should aim to disaggregate these conditions. Fourth, although CHARLS employs a multistage stratified probability-proportional-to-size sampling design, the present analysis did not incorporate the survey-provided sampling weights, stratification, or clustering variables; logistic regression models were therefore fitted to the analytic sample without adjustment for the complex survey design. As a result, the point estimates and their precision may not fully reflect the sampling structure of the original survey, and caution is warranted when generalizing these findings to the broader Chinese population of middle-aged and older adults. Future studies applying survey-weighted analyses using the CHARLS-provided weighting variables are needed to confirm the robustness of these associations.

### 4.4. Public health implications and future directions

Major preventable risk factors for CLDs include smoking, secondhand smoke, indoor air pollution, ambient particulate matter, ozone, and occupational exposures (e.g., coal dust) [83]. Interventions targeting these factors help reduce CLDs burden [4]. International efforts—such as tobacco control, air quality improvement, cleaner workplaces, allergen prevention, better management of comorbidities, and reducing underdiagnosis—have been implemented for decades [4]. Nevertheless, progress in reducing the overall CLDs burden remains limited, and CLDs continues to be a leading cause of disability and mortality worldwide [4,84].

In this context, our findings suggest that promoting physical activity may serve as an additional, low-cost, non-pharmacological component of healthy aging strategies [85]. Importantly, the observed associations were significantly modified by age and gender, and the protective link with higher-intensity activity was most evident among adults aged 65 and older and among male participants. Accordingly, public health policies and community programs in China might prioritize creating supportive environments (e.g., accessible spaces, group activities, simple messaging) that facilitate regular moderate-to-high intensity activity particularly for older adults and middle-aged men, while remaining inclusive for the broader middle-aged and older population. These directions align with the WHO 2020 guidelines on physical activity and sedentary behaviour [34] and are consistent with the U.S. Physical Activity Guidelines for Americans [86].

Future research should address the study’s limitations. Longitudinal analyses using multiple CHARLS waves are needed to clarify temporality and the sequence between physical activity and CLDs incidence. Studies incorporating objective measures (e.g., accelerometry for activity, spirometry for lung function) would strengthen validity. Randomized controlled trials remain the gold standard to assess whether structured physical activity interventions can prevent CLDs onset or slow progression in specific subgroups. Finally, further work should examine dose–response patterns and whether different domains of activity (leisure, occupational, transport) have differential relevance to lung health.

In conclusion, this cross-sectional study from a large nationally representative sample indicates that higher levels of physical activity are associated with lower CLDs prevalence among middle-aged and older adults in China, with the strongest evidence observed in adults aged 65 and older and in men. Although causality cannot be inferred from the current design, these findings support the value of promoting physically active lifestyles as part of public health strategies to reduce the burden of chronic disease in China and globally, with attention to demographic tailoring.

## 5. Conclusions

This cross-sectional analysis of nationally representative CHARLS data indicates a significant inverse association between physical activity and CLDs prevalence among Chinese adults aged 45 years and older, which is further modified by age and gender (P for interaction < 0.05). After adjusting for a range of potential confounders, higher levels of physical activity were associated with lower odds of CLDs, and stratified analyses suggested that this association was most evident among adults aged 65 and older and among male participants. In addition, moderate-intensity physical activity was also notably associated with reduced CLDs odds within the male subgroup. These findings support physical activity promotion as a potential public health strategy for CLDs prevention in China’ s aging population, with particular attention to older adults and middle-aged men. Future longitudinal studies and intervention trials are warranted to clarify temporal relationships, strengthen causal inference, and evaluate whether tailored physical activity interventions can reduce CLDs incidence or slow disease progression in specific demographic subgroups.

## Supporting information

S1 Appendix Collinearity diagnostics for all candidate independent variables considered in the multivariable logistic regression analysis of factors associated with CLDs. (DOCX)

## Abbreviations

CHARLS: China Health and Retirement Longitudinal Study
CLDs: Chronic lung diseases
COPD: Chronic obstructive pulmonary disease
BMI: Body mass index
MET: Metabolic equivalent
IPAQ: International Physical Activity Questionnaire
OR: Odds ratio
CI: Confidence interval
AIC: Akaike information criterion
VIF: Variance inflation factors
