# Supplementary material for: Associations between physical activity levels and chronic lung diseases in middle-aged and older adults in China: A CHARLS-based study
Source: PLoS One. 2026 Sep 8;21(9):e0354445. doi: 10.1371/journal.pone.0354445 (PMC13552760; doi:10.1371/journal.pone.0354445)
Supplement: Supplementary file 1 [file pone.0354445.s001.docx]

**S1 Appendix**

**Collinearity diagnostics for all candidate independent variables considered in the multivariable logistic regression analysis of factors associated with Chronic Lung Diseases (CLDs)**

*Note: Generalized Variance Inflation Factor (GVIF) and GVIF^(1/(2·Df)) are reported. For variables with Df = 1, GVIF equals the classical VIF. All GVIF^(1/(2·Df)) values were < 1.50, indicating no severe multicollinearity among the covariates.*

| **Variable** | **GVIF** | **Df** | **GVIF^(1/(2·Df))** |
| --- | --- | --- | --- |
| Exposure variable (physical activity level) | 1.052 | 2 | 1.013 |
| Gender | 2.134 | 1 | 1.461 |
| Residential area | 1.159 | 3 | 1.025 |
| Educational level | 1.195 | 2 | 1.045 |
| Smoking status | 2.001 | 1 | 1.414 |
| Drinking frequency | 1.200 | 2 | 1.047 |
| Hypertension | 1.143 | 1 | 1.069 |
| Hyperlipidemia | 1.206 | 1 | 1.098 |
| Diabetes | 1.140 | 1 | 1.068 |
| BMI | 1.004 | 1 | 1.002 |
| Waist circumference | 1.101 | 1 | 1.049 |

**Abbreviations:** *GVIF, Generalized Variance Inflation Factor; Df, degrees of freedom; BMI, body mass index.*

**Interpretation:** A common rule of thumb is that GVIF^(1/(2·Df)) > 2.0 indicates problematic multicollinearity. In this model, the highest value was 1.461 (gender), well below the threshold of 2.0, confirming that multicollinearity was not a concern in the multivariable logistic regression analysis.
